# Alteration of the Gut Microbiota and Its Effect on AMPK/NADPH Oxidase Signaling Pathway in 2K1C Rats

**DOI:** 10.1155/2019/8250619

**Published:** 2019-05-22

**Authors:** Hui Yu, Lei Qin, Hai Hu, Zhanli Wang

**Affiliations:** Department of Laboratory Medicine, The Second Affiliated Hospital, Baotou Medical College, Baotou 014030, China

## Abstract

**Background:**

The purpose of this study was to evaluate the alteration of the gut microbiota and its effect on adenosine monophosphate-activated protein kinase (AMPK)/nicotinamide adenine dinucleotide phosphate oxidase (NADPH oxidase) signaling pathway in two-kidney one-clip (2K1C) rats.

**Methods:**

The 2K1C rat models were established. The rats were randomly divided into the following 2 groups: 2K1C group and sham group. Alterations of the gut microbiota were analyzed based on the high throughput sequencing method. Plasma concentrations of short chain fatty acids (SCFAs) were measured by chromatography. The protein expression of phosphorylated AMPK and acetyl-CoA carboxylase (ACC) was determined by western blotting. NADPH oxidase activity was measured by a luminometer.

**Results:**

Microbial community analyses revealed that the structure and composition of the gut microbiota were significantly disrupted in 2K1C rats when compared to sham rats. This disruption was associated with the drastic increase in relative abundance of the genera* Prevotella* and the decrease in SCFA-producing bacterial population. We further confirm that SCFAs produced by the gut microbiota influence NADPH oxidase activity through AMPK.

**Conclusions:**

Our data implicated the important role of gut microbiota in the regulation of AMPK/NADPH oxidase signaling pathway.

## 1. Background

Hypertension has been recognized as a serious public health problem because it remains the most prevalent risk factor for cardiovascular disease [1]. Renovascular hypertension (RVH) represents a secondary form of hypertension resulting from atherosclerotic disease or fibromuscular dysplasia (FMD) of the renal arteries [2]. Although the pathophysiology of RVH is complex, the important causes involve the activation of the renin-angiotensin-aldosterone system (RAAS) and the presence of inflammation and oxidative stress responses [3, 4]. The 2K1C model is a long-established and widely employed model of hypertension in the study of RVH [5].

The intestine of an adult human is inhabited by diverse microorganisms, the diversity of which is estimated to be 36,000 bacterial species [6]. A growing body of evidence indicated that the gut microbiota exerted important influences on the development of hypertension [7, 8]. Researchers have identified multiple possible hypotheses to link dysbiosis and hypertension, such as modulation of endothelial-derived nitric oxide (NO) and chronic inflammation [9, 10]. Recently, many studies focused on the role of byproducts of gut microbial metabolism such as short chain fatty acids (SCFAs), which are generally thought to affect several molecular changes associated with improved cardiovascular health and function [11].

The adenosine monophosphate-activated protein kinase (AMPK)/nicotinamide adenine dinucleotide phosphate (NADPH) oxidase signaling pathway is involved in the inflammatory and oxidative stress responses and plays an important role in the regulation of cardiovascular function [12]. Growing evidence also suggested that gut microbiota regulated AMPK activity [13]. However, additional research is needed to determine whether the AMPK/NADPH oxidase signaling pathway is involved in the gut microbiota regulation of oxidative stress response in 2K1C rats.

The purpose of this study was to investigate whether intestinal microbes influence AMPK and NADPH oxidase activity through their metabolism SCFAs, providing a potential theoretical basis for a mechanism of endothelial dysfunction in 2K1C rats.

## 2. Methods

### 2.1. Animal Experiments

All procedures performed on rats were approved by the Institutional Animal Care and Use Committee. All operations were performed according to international guidelines concerning the care and treatment of experimental animals. Male Wistar rats aged 7 weeks (body weight, 160 to 180 g) purchased from Vital River Laboratory Animal Technology Co., Ltd. (Beijing, China) were cage housed and maintained in a temperature-controlled room with 12-hour light/dark cycles, with free access to water and standard rat chow. All rats were randomly divided into 2 groups: 2K1C group (n=8) and sham group (n=8). 2K1C model was then established as described in detail previously [14]. In briefly, rats were anaesthetized by intraperitoneal injection of pentobarbitone sodium (50 mg/kg body weight). The surgery was performed to implant a silver clip around the left renal artery. After the operation, the rats were housed individually. Body weight and blood pressure were assessed at the same time every week. For the noninvasive measurement of blood pressure, rats were placed in a warm incubator for 15 minutes. The tail-cuff plethysmography (Chengdu Instrument Factory, Sichuan, China) was connected in a quiet state to record the tail arterial pressure. The measurements were performed three times to calculate the average. At the week 8 after the operation, the animals are sacrificed under deep anesthesia for sample collection.

### 2.2. Fecal DNA Extraction, Metagenomic Sequencing, and Analysis

Fresh fecal contents were directly collected from the rat's cecum at the end of the study and stored in Sample Protector (TaKaRa, Dalian, China) at −80°C. The MoBio Power Fecal DNA Isolation kit (Mo BioLaboratories, Carlsbad, CA, USA) was used for DNA extraction. The quality of the extracted DNA was examined by agarose gel electrophoresis, and the OD 260/280 was analyzed by spectrophotometry. DNA libraries were prepared from 2 *μ*g of total DNA for each sample using TruSeq DNA LT Sample Prep Kit v2 (Illumina, San Diego, California). Metagenomic sequencing was performed on HiSeq 3000 platform (Illumina, San Diego, California). After removing adapters, the raw reads were filtered to remove low-quality reads and reads that belong to the host. These high-quality reads from the samples were then assembled to contigs using Meta-Velevt. MetaGeneMark was employed to predict open reading frames (ORFs). In addition, a metagenomic catalog was generated based on the samples obtained in this study. Furthermore, the high-quality clean paired-end reads from each sample were aligned by BWA version 0.5.7-6 to the reference genes. Then the relative abundance of genes was predicted as described previously [15].

### 2.3. Quantitative Polymerase Chain Reaction (qPCR)

Real-time qPCR analysis was carried out on ABI Prism 7500 (Applied Biosystems, California, USA) using Maxima SYBR Green/ROX qPCR Master Mix (2×) (Thermo Scientific, Massachusetts, USA). The primer sequences are showed in Table 1.

### 2.4. Measurement of Malondialdehyde (MDA) Concentration and Superoxide Dismutase (SOD) Activity

At the end of experiment, rats were sacrificed under deep anesthesia and thoracic aorta was quickly excised. After removing the adhering fat, aortic specimen was rinsed in a cool normal saline and was homogenized in a solution containing 250 mmol/l sucrose, 1 mmol/l EDTA, 0.1 mmo/l phenylmethylsulfonyl fluoride (PMSF), and 20 mmol/l potassium phosphate buffer at pH 7.6. The aorta homogenate was then centrifuged at 10,000 g for 5 min to collect the supernatant. The SOD activity and MDA content in the aorta homogenate were assayed using the commercial kits (Nanjing Jiancheng Bioengineering Institute, Nanjing, China).

### 2.5. SCFAs Extraction and Analysis

Plasma concentrations of SCFAs were measured as previously described [16]. Briefly, 100 *μ*l of plasma were diluted in acidified water spiked with stable isotope-labelled internal standards. Then, SCFA samples were extracted with 2 ml diethyl ether and were measured in an Agilent 6890 gas chromatography (GC) coupled with mass spectrometry (Agilent Technologies, Wilmington, DE, USA). The GC was equipped with a ZB-WAX column (Phenomenex, Cheshire, UK). For the measurement of fecal SCFAs levels, 100 mg fecal contents were mashed in acidic water (pH=2.4) and centrifuged at 12,000 g for 20 min at 4°C. The supernatants were then taken for analysis as previously described [17].

### 2.6. Western Blot Analysis

For western blotting analysis, tissue samples were homogenized in ice-cold cell lysis buffer (Beyotime, Shanghai, China) and the lysates were centrifuged at 12,000×g for 20 min at 4°C. The protein concentrations were determined by bicinchoninic acid (BCA) assay (Sigma-Aldrich, St. Louis, MO, USA). The protein samples were then subjected to gel electrophoresis and quantitative western blotting. The following antibodies were used: anti-AMPK, anti-p-AMPK^T172^, anti-acetyl-CoA carboxylase (ACC), anti-p-ACC^S79^, and anti-*β*-Actin (Abcam, Cambridge, UK). The specific bands were detected with an enhanced chemiluminescence detection system (Sage Creation, Beijing, China).

### 2.7. Cell Culture and Measurement of NADPH Oxidase Activity

HAECs (human aortic endothelial cells) were cultured in endothelial cell growth medium (EGM), which consists of EBM endothelial cell basal medium supplemented with the EBM-bullet kit reagents (Clonetics, Walkersville, MD, USA) and 2% fetal bovine serum. At confluence, HAECs were exposed to Angiotensin II (AngII, 200 nM) for 4 hours as described previously [18], and then stimulated by acetate (10 *μ*g/ml) in the presence or absence of Compound C (20 *μ*M, Sigma-Aldrich, St. Louis, MO, USA) for 10 min. For measurement of NADPH oxidase activity, cells were washed with ice-cold Hanks' balanced salt solution (HBSS) and lysed in cell lysis buffer (Cell Signaling Technology, Boston, MA, USA). The cell lysates were centrifuged for 10 min at 12,000 g. The lysates were then mixed with NADPH (100 *μ*M), lucigenin (5 *μΜ*), HBSS buffer, and water in 96-well plates and incubated for 30 min at 37°C. NADPH oxidase activity was measured by photon emission from the chromogenic substrate lucigenin in a luminometer (Berthold, NH, USA) at 450 nm wavelength. The levels were expressed as relative light units/min/mg of total protein.

### 2.8. Analysis of NO Level

Because of low concentrations and short half-life of NO in vivo, we determined the concentration of serum NO by measuring its stable metabolites nitrite (NO_2_^−^) and nitrate (NO_3_^−^) as described previously by Miranda et al. [19]. The concentration of NO in conditioned media of HAECs was estimated using a commercial kit (Nanjing Jiancheng Bioengineering Institute, Nanjing, China) in accordance with the manufacturer's instructions.

### 2.9. Statistical Analysis

Statistical analysis was performed with SPSS20.0 software. Data were analyzed for normal distribution with the independent t-test or paired t-test. The independent t-test was used in comparing between 2K1C group and sham group. Paired t-test was used in comparing the body weight, blood pressure and related knowledge on RVH before and after intervention. The values were expressed as mean ± standard error of the mean (SEM). Values of* P* < 0.05 were considered statistically significant.

## 3. Results

### 3.1. Establishment of 2K1C Hypertensive Rats

**Figure 1:**
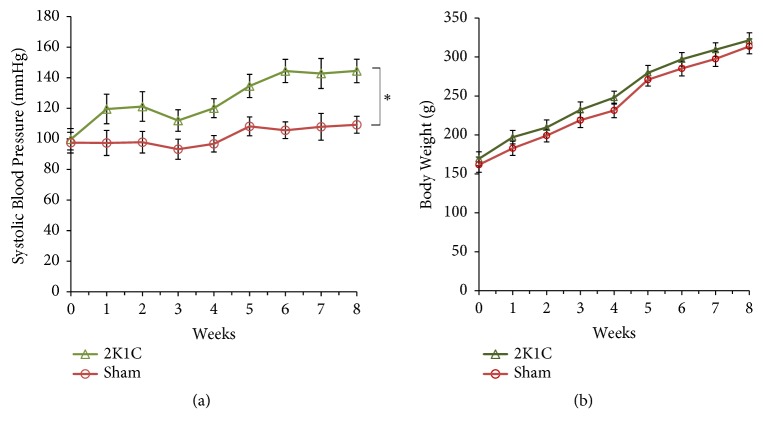
Establishment of 2K1C hypertensive rats. (a) SBP across experiment for 2K1C and sham-operated rats during a 8-week period. Values are expressed as the means ± SE, n = 8 rats per group. *∗P*<0.05,* vs* sham group. (b) Body weight of the animals from the different experimental groups. Values are expressed as the means ± SE, n = 8 rats per group. *∗P*<0.05* vs* sham group.

Firstly, we developed and evaluated 2K1C model. As showed in Figure 1(a) the systolic blood pressure (SBP) was significantly increased in 2K1C rats when compared with sham group (*P*<0.05). Body weight is presented in Figure 1(b). There was no significant difference among the groups.

### 3.2. Taxonomy-Based Comparisons of Gut Microbiota at the Phylum and Genus Levels

**Figure 2:**
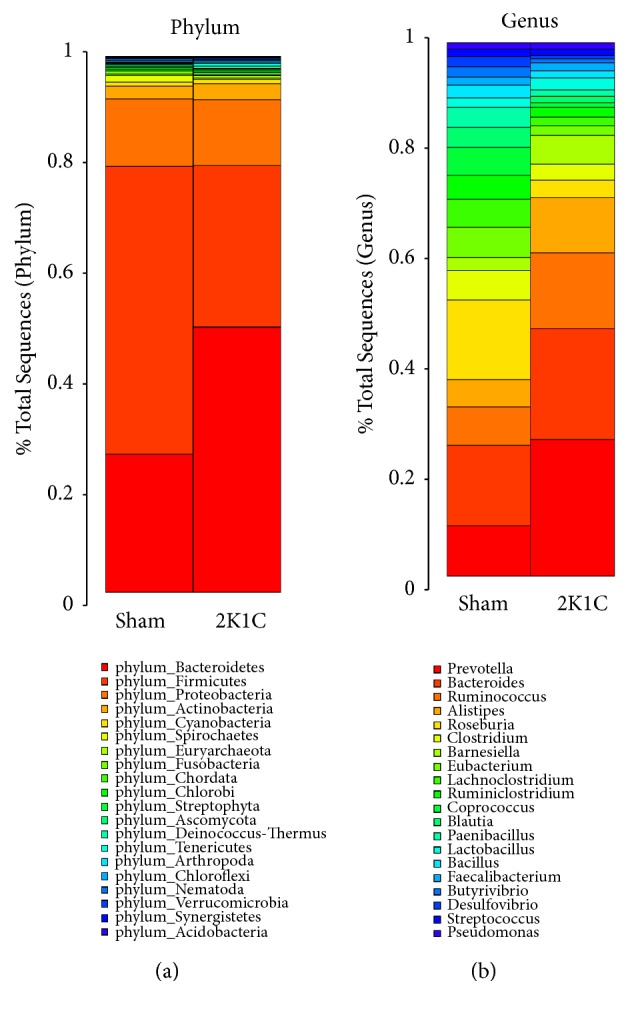
The relative abundance of microbial species in 2K1C rats (n=8) and sham rats (n=8) fecal samples. (a) Taxonomic summary of the gut microbiota at phylum level. (b) Taxonomic summary of the gut microbiota at genus level.

As showed in Figure 2(a), the composition of gut microbiota of all rats was mainly characterized by the phyla* Bacteroidetes*,* Firmicutes*,* Proteobacteria*,* Actinobacteria*, and* Cyanobacteria*. There were marked differences in the distribution of phyla between two groups. These results showed a reduction in* Firmicutes* and an increase in* Bacteroidetes* in 2K1C rats compared to sham controls (*P*<0.05). However, there were no significant differences in* Proteobacteria*,* Actinobacteria*, and* Cyanobacteria* between two groups (*P*>0.05) (Figure 2(a)).

We next analyzed the differences in the distribution of genus between 2K1C and sham groups (Figure 2(b)). Increased abundance of the genera* Prevotella*,* Bacteroides*,* Alistipes*, and* Barnesiella* within the* Bacteroidetes* phylum was detected (*P*<0.05) in 2K1C rats. Within the* Firmicutes* phylum, the abundance of the genera* Lactobacillus* and* Ruminococcus* was increased (*P*<0.05), but* Coprococcus*,* Roseburia*,* Blautia*,* Clostridium*,* Eubacterium*,* Lachnoclostridium*,* Ruminiclostridium*,* Paenibacillus*,* Bacillus,* and* Butyrivibrio* were decreased (*P*<0.05). Additionally, the relative abundance of* Desulfovibrio* genus within the* Proteobacteria* phylum was significantly lower in 2K1C rats than that in sham controls (*P*<0.05). However, the abundance of the genera* Streptococcus* and* Faecalibacterium* within the* Firmicutes* phylum and the genus* Pseudomonas* within the* Proteobacteria* phylum did not differ significantly between two groups (*P*>0.05).

**Figure 3:**
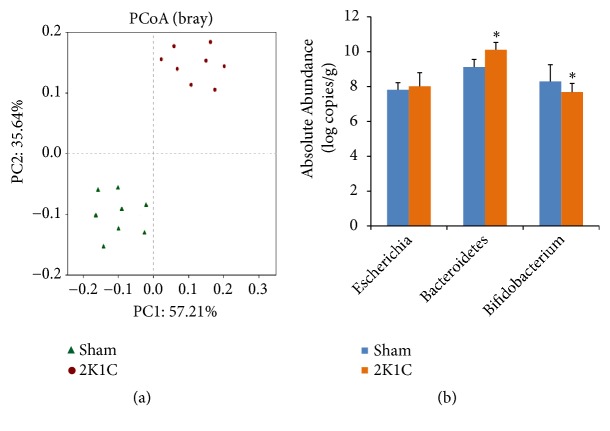
Compositional and structural shifts of gut microbiota in 2K1C rats (n=8) and sham rats (n=8) fecal samples. (a) PCoA analysis of gut microbiota from 2K1C rats and sham rats. (b) Quantitative PCR analysis of three representative bacteria. Values are expressed as the means ± SE. *∗P*<0.05* vs* sham group.

The results of principal coordinate analysis (PCoA) revealed major shifts in the gut microbiota composition between two groups (Figure 3(a)). Moreover, qPCR data showed significant increases of* Bacteroidetes *spp. and reduction of* Bifidobacterium *spp. in 2K1C group compared to sham group (*P*<0.05) (Figure 3(b)). However, no obvious difference was found in* Escherichia *spp. population (*P*>0.05).

### 3.3. Measurement the Levels of SOD, MDA, and NO

**Figure 4:**
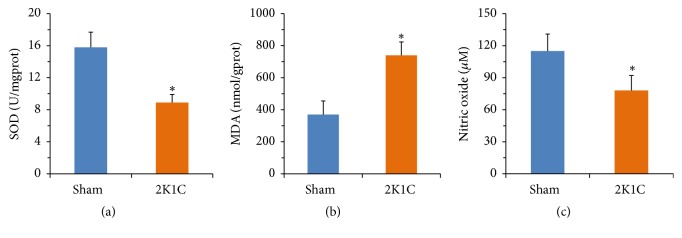
The levels of SOD, MDA, and NO in rats. Values are expressed as the means ± SE, n = 8 rats per group. *∗P*<0.05* vs* sham group.

The SOD activity was decreased in aorta of 2K1C rats when compared with sham rats (*P*<0.05) (Figure 4(a)). However, the MDA level was significantly enhanced in 2K1C rats (*P*<0.05) (Figure 4(b)). The serum NO measurements are presented in Figure 4(c). The serum NO concentration in 2K1C rats was significantly lower than sham group (*P*<0.05).

### 3.4. Measurement the Levels of Plasma SCFAs

**Figure 5:**
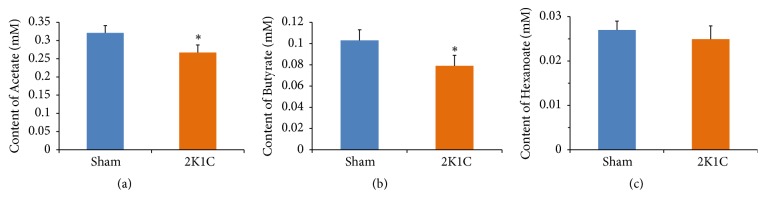
Plasma SCFAs levels in rats. Values are expressed as the means ± SE, n = 8 rats per group. *∗P*<0.05* vs* sham group.

Compared with sham rats, plasma levels of acetate and butyrate were significantly decreased in 2K1C rats (*P*<0.05) (Figures 5(a) and 5(b)), suggesting that differential bacterial community affects plasma SCFA levels. Hexanoate was not different between two groups (*P*>0.05) (Figure 5(c)). Similarly, fecal levels of acetate and butyrate were significantly decreased in 2K1C rats compared with sham rats (*P*<0.05) (Figure S1).

### 3.5. Measurement the Phosphorylation Levels of AMPK

**Figure 6:**
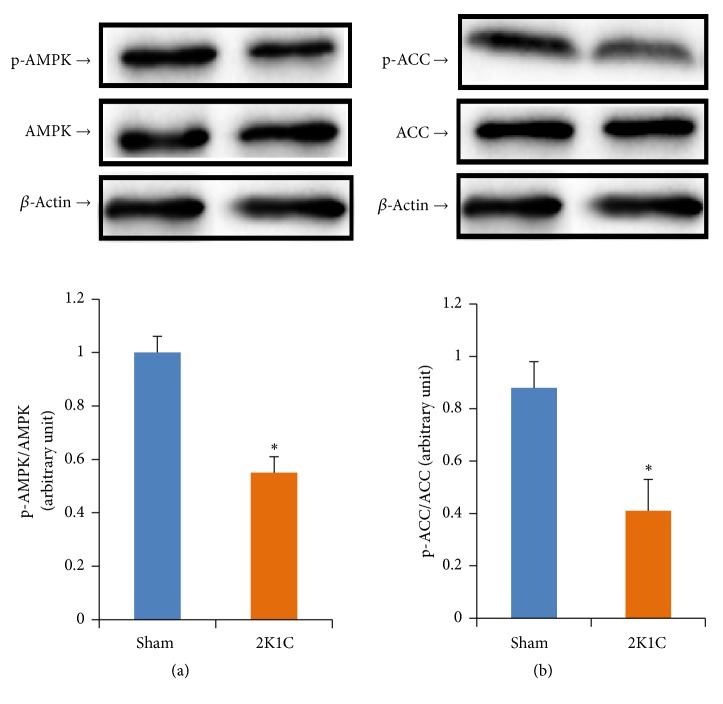
The phosphorylation levels of (a) AMPK and (b) ACC in aortas. Values are expressed as the means ± SE, n = 8 rats per group. *∗P*<0.05* vs* sham group.

Western blot analysis demonstrated that AMPK and ACC phosphorylations were significantly attenuated in 2K1C rats (*P*<0.05) (Figure 6). However, the expressions of total AMPK and ACC were not altered (*P*>0.05) (Figure 6).

### 3.6. Acetate Affected NADPH Oxidases Activity and NO Secretion via AMPK

**Figure 7:**
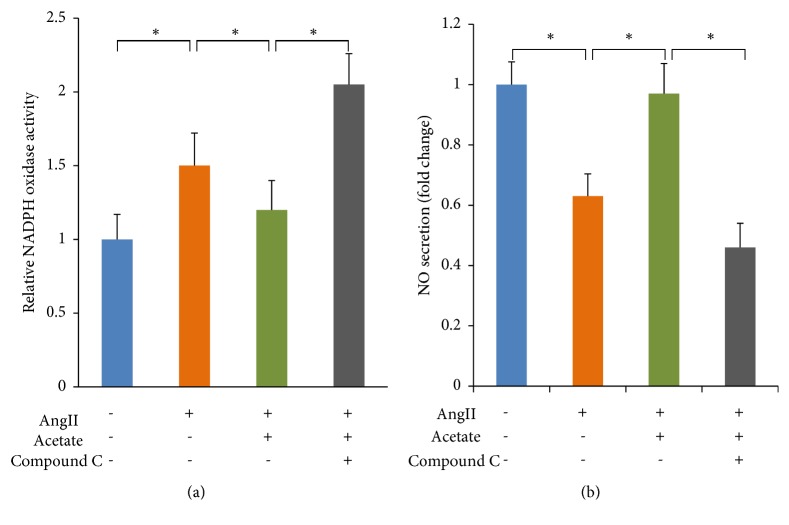
The effects of acetate on the activity of NADPH oxidase in HAECs and the level of NO in conditioned media of HAECs. (a) Effect of acetate on the activity of NADPH oxidase. (b) Effect of acetate on the level of NO. *∗P*<0.05* vs* sham group.

Figure 7(a) showed that acetate significantly decreased NADPH oxidases activity (*P*<0.05). Inhibition of AMPK blocked the acetate-induced decrease in NADPH oxidases activity.  Figure 7(b) showed that the NO level was increased significantly in acetate-treated HAECs (*P*<0.05). Similarly, the acetate-induced increase in NO secretion was abolished by Compound C, indicating that this effect of acetate was mediated by AMPK.

## 4. Discussion

The involvement of gut microbiota in regulating hypertension is unequivocally established in the literature [20]. However, to our knowledge, there are no gut microbiotal studies reported in 2K1C models.

Our study provided the first evidence of an association of RVH with altered gut microbiota with the use of 2K1C model. In the present study, gut microbiota composition and diversity were analyzed based on the high throughput sequencing method.* Firmicutes* and* Bacteroidetes* were the most dominant among the 20 phyla in the samples, which is consistent with the previous reports [21]. In order to further study the alterations of the gut microbiota in 2K1C model, taxonomy-based comparisons of gut microbiota at the genus level were explored. Interestingly, drastic increase in the relative abundance of the genera* Prevotella* within the* Bacteroidetes* phylum was detected in 2K1C rats (18.2%) when compared with control group (2.9%). Previous studies have shown that the genera* Prevotella* is more abundant in the intestinal tract of healthy people [22]. However, many recent studies have also suggested that the genera* Prevotella* may be involved in the pathogenesis of hypertension. Li* et al*. found* Prevotella*-dominated gut enterotype and overgrowth of* Prevotella* in both prehypertensive and hypertensive populations compared to healthy controls, suggesting that* Prevotella* contributes to the development of hypertension [23]. Our data corroborate these findings.

We further found that structural shift of gut microbiota in 2K1C rats was also associated with decrease in acetate-, butyrate-producing microbial populations, including* Roseburia*,* Clostridium*,* Eubacterium*,* Blautia*,* Butyrivibrio*,* Bacillus*, and* Coprococcus*. Consistent with the above results, both acetate and butyrate levels were lower in 2K1C group, which was accompanied by increased level of MDA, and decreased activity of SOD. It has been reported that SOD activity and MDA content were attributed to the endothelial function [24].

As the end products of fermentation by the gut microbiota, SCFAs play critical roles in maintaining a healthy cardiovascular function [25]. Recently, Ganesh et al. also reported that acetate plays a key role in the regulation of blood pressure [26]. In order to determine whether SCFAs participate in the regulation of oxidative stress status in 2K1C rats, we assessed the effects of acetate (representing the SCFA) on the activity of NADPH oxidase. Our results showed that acetate significantly decreased NADPH oxidase activity. More importantly, these effects of acetate were abolished by Compound C, an AMPK inhibitor, indicating that acetate decreased NADPH oxidase activity via activation of AMPK. Consistently, we found that acetate induced increase of the NO level in an AMPK-dependent manner. It is well known that the increase of NADPH oxidase activity impairs vascular endothelial function through superoxide (O_2_^−^) production [27]. Thus, our results provide the necessary basis to further examine relationship between the alteration of gut microbiota and endothelial dysfunction in 2K1C rats.

## 5. Limitations

The current study also has some limitations. First, the overall activity of NADPH oxidase activity but not the activity of each NADPH oxidase isoform was measured in this study. Activation of different NADPH oxidase isoforms might have different effects on endothelial function. Thus, further research is needed to investigate the activity of each NADPH oxidase isoform in both 2K1C rats and HAECs. Second, SCFAs derived from both bacterial fermentation and other different sources. Therefore, the respective roles and interactions of gut microbiota and SCFAs in 2K1C rats need to be further clarified. Third, AMPK can be activated by entirely different events. The deeper signaling mechanisms that SCFAs regulate AMPK/NADPH oxidase signaling pathway are still needed to be demonstrated by further studies.

## 6. Conclusions

The results of this study demonstrated the diversity of gut microbiota and its important role in the regulation of AMPK/NADPH oxidase signaling pathway in 2K1C rats. Although additional study is needed to validate this signaling pathway, the results provide new insights into the theoretic basis of gut microbiota-mediated regulation of endothelial dysfunction.

## Figures and Tables

**Table 1:** Primers used for real-time PCR analyses.

Target group	Sequence
*Escherichia*	F-GTTAATACCTTTGCTCATTGA
	R-ACCAGGGTATCTAATCCTGTT
*Bifidobacterium*	F-CGCGTCYGGTGTGAAAG
	R-CCCCACATCCAGCATCCA
*Bacteroidetes*	F-AGCAGTAGGGAATCTTCCA
	R-CACCGCTACACATGGAG

## Data Availability

All data generated or analyzed during this study are included in this published article or available from the corresponding author upon reasonable request.

## Abbreviations

AAA: Abdominal aortic aneurysm
ACC: Acetyl-CoA carboxylase
AMPK: Adenosine monophosphate-activated protein kinase
AngII: Angiotensin II
BCA: Bicinchoninic acid
EGM: Endothelial cell growth medium
FMD: Fibromuscular dysplasia
GC: Gas chromatography
HAEC: Human aortic endothelial cell
HBSS: Hanks' balanced salt solution
MDA: Malondialdehyde
NADPH: Nicotinamide adenine dinucleotide phosphate
NO: Nitric oxide
ORF: Open reading frame
PCoA: Principal coordinate analysis
PMSF: Phenylmethylsulfonyl fluoride
qPCR: Quantitative polymerase chain reaction
RAAS: Renin-angiotensin-aldosterone system
RVH: Renovascular hypertension
SBP: Systolic blood pressure
SCFA: Short chain fatty acid
SOD: Superoxide dismutase
2K1C: Two-kidney one-clip.

## Supplementary Materials

Supplementary Materials Figure S1: fecal SCFAs levels in rats. Values are expressed as the means ± SE, n = 8 rats per group. *∗*P<0.05 vs sham group.
